# Role of Helicobacter pylori in Gastric Carcinoma: A Review

**DOI:** 10.7759/cureus.37205

**Published:** 2023-04-06

**Authors:** Anukriti Kesharwani, Onkar R Dighe, Yashwant Lamture

**Affiliations:** 1 Department of General Surgery, Jawaharlal Nehru Medical College, Datta Meghe Institute of Higher Education and Research, Wardha, IND

**Keywords:** dysplasia, metaplasia, adenocarcinoma, gastric tumour, microbes, gastric cancer, helicobacter pylori

## Abstract

Gastric cancer (GC) is one of the leading causes of cancer-related deaths globally. Gastritis caused by* Helicobacter pylori (H. pylori)* is a potent cause of gastrointestinal malignancies. The majority of all humans on the planet have *H. pylori *invasion in their stomachs, yet only a few diseased people develop GC. The human gastrointestinal system contains a broad population of microorganisms in addition to *H. pylori.* *H. pylori *heterogeneity has been studied because not all *H. pylori* diseases result in cancer. Individuals in the adult age group account for the bulk of gastric carcinoma cases. *H. pylori* has various strains, which is beneficial for its survival in host cell epithelium for a longer duration of time. Along with *H. pylori*, oral microbes have a major role in the pathogenicity of gastric carcinoma. The complex ecology of oral microbiota helps to defend against infections, preserve homeostasis, and regulate the immune system. In contrast, oral microbiota is involved in various mechanisms like anti-apoptotic activity, suppression of the immune system of the host, and initiation of chronic inflammation. These oral microbes are also responsible for the development of mutations. Interactions between the host immune system and bacteria promote the progression of cancer. For this review, various research articles were studied, and information was collected using databases like PubMed and Google Scholar. This review emphasizes on the role of *H. pylori* in gastric carcinoma, its pathogenesis*,* the role of various virulence factors and risk factors related to it, the role of oral microbiota in gastric carcinoma pathogenesis, diagnostic modalities, treatment options, and preventive measures for gastric carcinoma.

## Introduction and background

*Helicobacter pylori* *(H. pylori)* is a gram-negative flagellated bacterium. It is highly diversified, and it has evolved to flourish in the stomach niche [1]. *H. pylori *is the most abundant bacterium in the stomach microbiota, accounting for 40 to 90 percent of the total [2-3]. It has chronic colonization in 50 percent of the world's population, and approximately 15 percent of those with the infection develop gastric cancers [4]. Helicobacter infection is a major contributor to stomach carcinoma in the world. Around 75 percent of all non-cardia cancers and 63 percent of all gastric cancer (GC) worldwide are due to *H. pylori* [5]. It is also responsible for the development of duodenal ulcers and type B gastritis. Infection by this bacterium elicits a chronic active immunological response that endures throughout the host's lifetime without antibiotic-induced eradication. Each strain of *H. pylori* exhibits changes in its genome sequence of more than 20 percent due to the plasticity of the genome [6]. As a result, during long-term colonization, even a single strain may produce a variety of variations and adapt to a particular host environment. Communities affected with* H. pylori* have more chances of growing GC than communities without the disease. Sometimes people affected with *H. pylori *infection are asymptomatic, and later, they develop peptic ulcers and GC. Most stomach cancer cases are advanced-type when they first manifest [7]. The prognosis is bad for these instances because roughly 40 percent of patients don't manifest cancer symptoms before getting confirmation. Oral microbes also have a correlation with the development of gastric carcinoma. These microbes are responsible for carcinogenic activity due to their various mechanism like suppression of the immune system of the host and the development of anti-apoptotic activity in cells. Other risk factors responsible for gastric carcinogenesis are family history, excessive salt intake, smoking, and oxidative stress. Preventive measures for gastric cancer include behavioral changes, dietary interventions, maintenance of healthy weight, caseation of smoking, and alcohol abstinence [8]. We have thoroughly discussed all these mentioned aspects in this review. 

## Review

Methodology 

**Figure 1:**
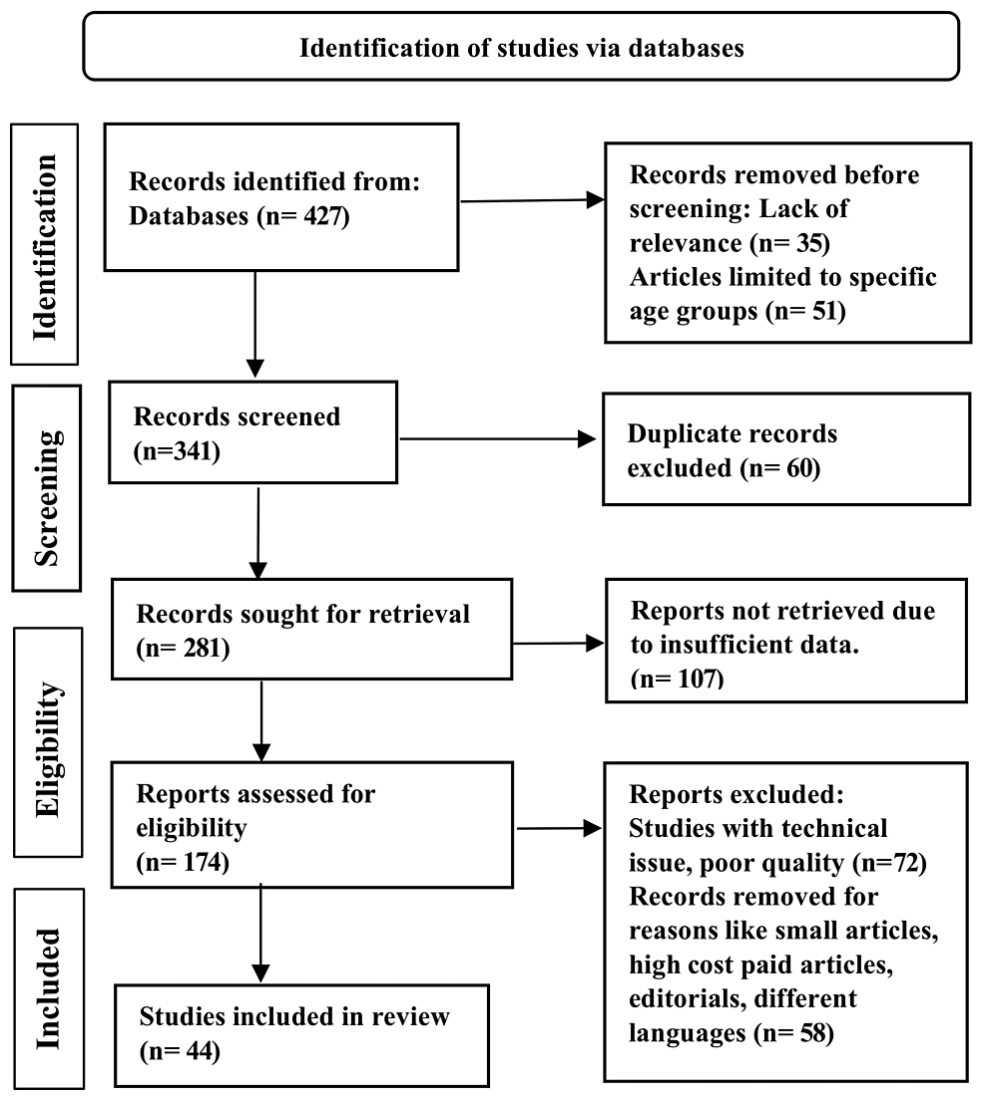
PRISMA model for the search strategy PRISMA: Preferred Reporting Items for Systemic Reviews and Meta-Analysis

Advanced PubMed search, Embase, and Google Scholar were used to search the following keywords: dysplasia and metaplasia and adenocarcinoma and gastric carcinoma, and *helicobacter pylori*. A total of 427 articles were found through the search, and 44 were chosen for study. The methodology by Preferred Reporting Items for Systemic Reviews and Meta-Analyses (PRISMA) method is shown in Figure 1 below.

Cancer stem cells and gastric carcinoma

Cancer can be seen as a complex structure made up of several cell types at different stages of development and varying capacities for proliferation. A tumor stem cell is a population of tumor cells that serve as the source of cancer cells [8]. The idea that tumor stem cells are related to peripheral stem cells is strengthened by the fact that these peculiar cells share many similar characteristics. The ability of peripheral stem cells to momentarily override average growth regulatory mechanisms, growth of tissue regeneration, and the ability to grow in the presence of contradictory signals is well known to raise the risk of mutation. The discovery is that the region where the stem cells are considered to be located is frequently harmed and depleted by substances thought to cause cancer. Persistent inflammation, which affects more than just the stomach, typically causes degeneration and the loss of specialized cells in other organs. These changes act as an early stage in the growth of invasive cancer. Although the molecular mechanisms underlying stomach carcinogenesis are not fully understood, various factors, including genetic profile and lifestyle choices, have been linked to the emergence of gastric cancer (GC) [9].

Association of *Helicobacter pylori* in the pathogenesis of gastric carcinoma

*Helicobacter pylori (H. pylori)* is the main factor responsible for stomach cancer pathogenesis. Every population has been proven to be predisposed to it. Being overweight and having gastro-esophageal reflux disease are all risk factors for GC [10]. The coexistence of a genetically susceptible host, a bacterial virulence strain, and a sensitized stomach habitat may contribute to cancer development. *H. pylori* have different enzymes to tackle the acidic nature of the stomach. The severity of infection is determined by environmental factors as well as microbes and host characteristics [11]. Stomach carcinogenesis is a multistage process that can be reversed in the early stages of mucosal injury, but the precise point of no return has yet to be determined [12]. The infection causes chronic stomach inflammation and the destruction of the stomach's hydrochloric acid-secreting glands. Based on animal research, clinical monitoring, and human interventional studies, *H. pylori *is the leading risk factor for GC [13-14]. Some clinical manifestations of *H. pylori* are persistent gastritis with no symptoms, duodenal ulcers, and gastric ulcers. Some individuals may develop mucosa-associated lymphoid tissue (MALT) lymphoma [15].

Classification of gastric carcinomas

Gastric carcinoma includes proximal cancers and distal cancers. The proximal type differs from the distal type in their demographic as well as pathophysiology characteristics [16]. Most stomach cancers in the globe originate from the distal stomach, despite an increase in the frequency of proximal GC. *H. pylori* continues to play a significant part in the formation of stomach carcinogenesis. Another classification includes two types of stomach cancer, intestinal type of gastric carcinoma and diffuse type of gastric carcinoma [17]. The primary cause of intestinal type is *H. pylori* infection. Stomach cancer is more likely to affect men than women. Still, this difference is less noticeable now because the incidence of intestinal variety, which is more prevalent in men, is declining. There is an involvement of glands in the intestinal type, but no such involvement is seen in the diffuse type. The older age group is more affected by the intestinal type of carcinoma than the young population. Early-stage diffuse type of carcinoma commonly spreads into the submucosa and invades the higher layers of the stomach wall rather than developing into a tumor that protrudes into the lumen. Table 1 summarizes the types of carcinoma.

**Table 1:** Types of stomach carcinoma ECL: enterchromaffin-like cells The table is created from information from Waldum et al.'s article [17]

Characteristics	Intestinal type	Diffuse type
Involvement of glands	Yes	No
Transition from one to other	No	No
Macroscopic growth pattern	Spread of tumor into the lumen	Spread of tumor along the mucosa
Presence of e-cadherin	Seldom	Often
Origin of cells	Stem cell	ECL cell
Affected age group	Older	Young

Virulence factors related to gastric carcinoma

Numerous virulence factors found in *H. pylori* could interfere with the host's intercellular signaling cascades. The most important pathogenic components of virulence are cytotoxin-associated gene A (cagA), its pathogenicity island (cag PAI), vacuolating cytotoxin A (vacA), and bacterial flagellum [18].

Cag PAI and CagA

The cag PAI provides the most accurate assessment of *helicobacter* pathogenicity. A strain carrying cag PAI has more chance of producing distal GC than strains missing the cag island. Compared to cagA-negative strains, cagA-positive strains have more severe inflammatory reactions, higher levels of atrophy, and a greater likelihood of developing into stomach carcinoma [19]. Type IV secretion system (T4SS) is transcribed by the cag PAI, which secretes at least 18 proteins into host tissue, including cagA. After attaching with bacteria, cagA is moved into the host cells by T4SS. CagA undergoes tyrosine phosphorylation as soon as it enters the host cell, where it binds to the inner surface of the cell membrane. This affects multiple signal transduction pathways inside the cell and modifies the structure of the cell. CagA also has a pathogenic effect without being phosphorylated [20].

VacA

Each *H. pylori* strain has the vacA gene [21]. It performs various tasks, including opening a membrane gap and releasing cytochrome C from mitochondria to cause death. There is evidence from numerous studies that variations in the vacA gene structure are related to the severity of the clinical condition and variations in the signal region (s1 and s2), middle region (m1 and m2), an intermediate region (i1 and i2) [22]. According to studies, people in Africa and the Middle East who are infected with the vacA s1 or m1 strains are more likely to develop stomach and peptic tumors than people who are infected with the s2 or m2 strains [23].

*Flagellum* 

A key component permitting bacterial movement and chemotaxis capabilities is the *H. pylori* flagellum. According to its physiological makeup, *H. pylori* has two to six sheathed unipolar flagella that provide protection against the acidic microenvironment of the stomach. The precise number of flagella is related to the movement speed of *H. pylori* and may vary between species. Because the proton motive force is specifically generated by urease-driven hydrolysis, it may help to explain why bacteria migrate toward urea when they are chemotactically attracted to it [24]. One of the most important virulence factors associated with *H. pylori* pathogenicity is the flagellum, which promotes bacterial movement and helps to adapt in the gastric mucosa, especially in the early phases of bacterial invasion. Less mobile strains of *H. pylori* have been shown to have difficulty adapting to the stomach mucosa. Bacterial infectivity also depends upon bacterial mobility. The inability of non-flagellated *H. pylori* mutants to adapt to the gastric mucosa was demonstrated, and it reveals that bacterial motility is of utmost essential for continued bacterial pathogenicity. The chemotaxis pathway regulates bacterial movement and its flagellar motility. Additionally, it is thought that flagella play a role in biofilm development, immunological infiltration, and inflammation brought on by *H. pylori *[24].

Mechanism of oral microbiota in gastric carcinoma

The composition of oral microbes has been linked to the carcinogenesis of multiple organs, particularly the gastrointestinal tract. It is found that oral microbiota plays an important role in GC [25]. Modifications in the amount of oral flora can affect the maintenance of the local microenvironment, which has been connected to the growth of GC [26]. Oral microbes have many possible modes of action that could lead to cancer development. Various mechanisms are explained below.

Initiation of Chronic Inflammation

The most important preventable cause of gastric carcinoma is chronic inflammation. This inflammation also fastens the process of invasion of cancer and metastasis [27]. Mediators generated by oral microbes lead to DNA injury, arrest of the cell cycle, increased number of cells, the spread of a tumor, metastasis, and new blood vessel formation. Anaerobic bacterial species like *Porphyromonas, Fusobacterium,* and *Prevotella *are* *mainly responsible for chronic inflammation. These bacteria harm epithelial cells and extracellular matrix constituents while promoting the generation of inflammatory mediators [28].

Immune System Suppression in the Host

In the human gut, a large number of bacterial species are present. Bacterial interactions with the host immune system promote the progression of cancer. Bacterial species like *Porphyromonas gingivalis* and *Fusobacter nucleatum *enhance the inhibition of the host immune response and anti-oxidative systems of the host [29].

Carcinogenic Substances

Microbes in the mouth produce substances that contribute to chronic inflammation, mutation buildup, and cancer progression. Sulfur compounds, reactive nitrogen and oxygen species, hydrogen peroxide, and acids in organic form are substances produced by oral bacteria that have a cancer-causing effect. The microorganisms that convert alcohol to acetaldehyde substantially impact the development of cancer because they have the enzyme alcohol dehydrogenase, which is responsible for carcinogenesis [30].

*Anti-Apoptotic Activity * 

**Figure 2:**
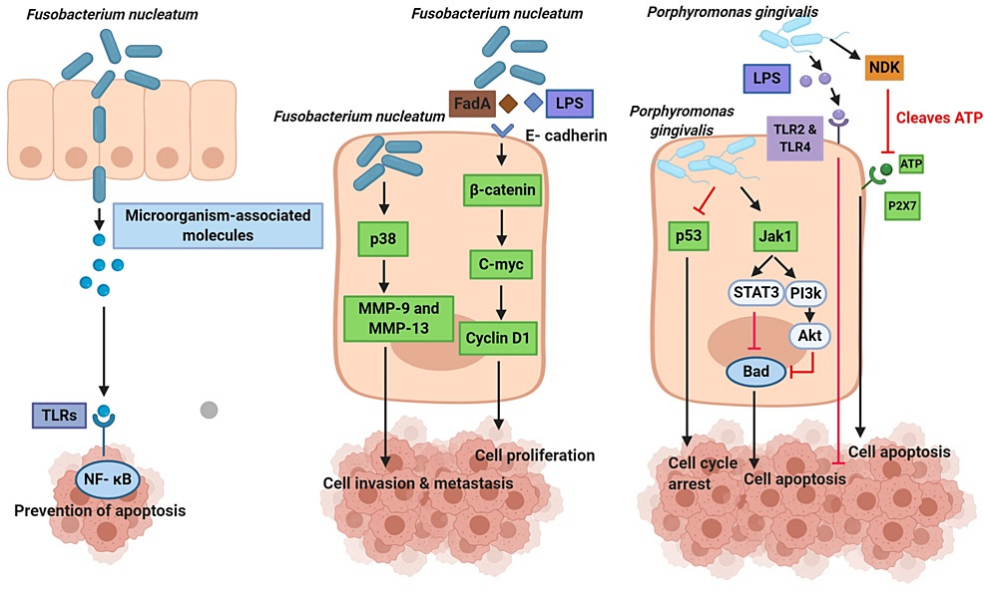
Mechanism of oral microbiota in inhibition of cell apoptosis. P2X7: purinergic receptor; ATP: adenosine triphosphate; NDK: nucleoside diphosphate kinase; CDK: cyclin-dependent kinase; Bad: Bcl-2-associated death promoter; p53: tumor protein p53; Akt: protein kinase B Stat3: signal transducer and activator of transcription 3; NF- kB: nuclear factor kappa B; p38: mitogen-activated protein kinase p38; LPS: lipopolisaccharide; Jak1: janus kinase 1; ; TLR: toll-like receptor; MMPs: matrix metalloproteinases; FadA: fusobacterial adhesin/invasin The author has taken the figure from an open-source journal under Open access journal which is under the Creative Commons Attribution 4.0 International License. Source: Bakhti et al. [26]

The stimulation of anti-apoptotic signaling pathways by oral microbes promotes tumor growth and suppression of pro-apoptotic pathways. It ultimately leads to the suppression of cellular apoptosis. *Porphyromonas gingivalis* and *Fusobacter nucleatum* (*F. nucleatum*) infection activate anti-apoptotic pathways, which inhibit cellular apoptosis and help in tumor growth. The infection with *F. nucleatum* alters a variety of anti-apoptotic mechanisms like activation of Toll-like receptors (TLR) and increases NF-kB signaling. It also activates p38, which causes the release of matrix metalloproteinases MMP-13 and MMP-9 and has a major impact on the invasion and metastasis of cancer cells. Beta-catenin signaling is induced by *F. nucleatum* by its lipopolysaccharide (LPS). *P. gingivalis* LPS initiates host response via TLRs, such as TLR4 and TLR2, which may inhibit apoptosis and promote tumor growth; as a result, it contributes to the survival of tumor cells and the development of cancer. *P. gingivalis *inhibits apoptosis by modulating several pathways. It intracellularly stimulates Jak1/Akt/Stat3 anti-apoptotic signaling [26]. Figure 2 summarizes the mechanism of oral microbiota in the inhibition of cell apoptosis.

Other risk factors

Family History 

It is another main risk factor related to GC. The majority of cases are sporadic, and only around some exhibit familial aggregation. People who have a genetic predisposition are almost three times more susceptible than others. People with a CagA-positive *H. pylori* infection and a family history of GC are more likely to acquire GC than those who are uninfected and have no family history of the disease [31-32].

Interleukin-1 (IL-1) Gene Cluster Polymorphism 

There is a direct connection between proinflammatory IL-1 gene cluster mutations and a severe inflammatory response that results in a deficiency of stomach acid and a higher chance of cancer [33]. In people with the interleukin-1 beta (IL-1B-31*C) and interleukin-1 receptor antagonist gene (IL-1RN*2/*2), genomes are more prone to develop stomach atrophy, stomach cancer, or hypochlorhydria brought on by *H. pylori* infection [34]. Compared to non-inflammatory genotypes, the high chance of developing cancer with these genotypes was two to threefold. These results have been verified in Caucasian, Hispanic, and Asian populations [35-36].

Salt Intake and Smoking

Salt intake also impacts the development of GC brought on by *H. pylori.* According to research conducted in Japan, increased nutritional salt consumption enhances the carcinogenic consequences of *H. pylori* strains that are cagA-positive [37]. Salt can harm gastric mucosa, causing inflammation, and also increasing the production of proinflammatory cytokines and enzymes like cyclooxygenase-2 (COX-2). Nitric oxide synthase may be linked to a high salt intake [38]. The most significant risk factor associated with lifestyle is smoking. Smoking is related to an enormously increased relative risk for both GC and malignancies [39].

Oxidative Stress 

Potentially harmful elements that contribute to the stomach mucosal lesion caused by *H. pylori* include oxygen-derived free radicals released by active neutrophils. Neutrophils express myeloperoxidase, the enzyme responsible for producing the hypochlorous anion. Urease is the most abundantly expressed protein by *H. pylori.* Urease is thought to stimulate angiogenesis, resulting in faster GC progression. Greater urease activity may be linked to an increased risk of inducing histopathological changes in the gastric mucosa and further gastric carcinogenesis. Urea is converted into ammonia by the *H. pylori-*associated urease, which yields monochloramine. This lipophilic and extremely hazardous oxidant easily crosses biological membranes and oxidizes components inside the cell [40]. Free radicals can attach to nucleic acids and cause mutations contributing to multistep carcinogenesis [41].

Diagnostic tests

H. pylori Diagnosis

Various tests like the urea breath test, stool antigen testing, and *H. pylori* antibody testing are used to diagnose the disease and to check whether treatment is working or not. Due to their speed and lack of invasiveness, these tests are the most often carried out [42].

Urea Breath Test

A low concentration of nonradioactive or radioactive material is present in a solution that is consumed by a patient. The substance is converted into labeled carbon dioxide gas which is released in the breath if the person has *H. pylori* in their digestive tract [42].

H. pylori Antibody Testing

This test does not discriminate between a past infection and a current infection and detects antibodies to the bacterium. If this test is positive, then other confirmatory tests are done to make a final diagnosis [42].

Gastric Cancer Diagnosis

After an *H. pylori* infection, if there is a suspicion of gastric cancer, then various diagnostic imaging studies are required for confirmatory diagnosis. Some studies are non-invasive, like X-ray, magnetic resonance imaging (MRI), computed tomography (CT) scan, endoscopic ultrasound, and positron emission tomography (PET) scan. Invasive tests include upper endoscopy with biopsy and laparoscopy [42].

Imaging Studies: Endoscopic Ultrasound, CT Scan, MRI, and PET Scan

Endoscopic ultrasonography is frequently used to determine how far a tumor has grown into the stomach wall, adjacent tissues, or surrounding lymph nodes. The stomach can be seen well on a CT scan, which can also confirm the presence of a malignancy. The liver and nearby lymph nodes are two additional locations that a CT scan can reveal that may have been affected by the spread of stomach cancer. This can assist in evaluating the progression of cancer. MRI gives images of the body's soft tissues, much like a CT scan does. A pET scan is useful for detecting the progression of cancer. During this test, patients receive an injection of sugar that is somewhat radioactive and concentrates mostly in cancer cells. Afterward, a photograph of the radioactive regions is made using a specific camera. PET scans detect potential areas of cancer spread in the body [42].

Upper Gastrointestinal (GI) Series

The patient consumes a barium-containing white, chalky fluid for this test. The esophagus, stomach, and small intestine are coated with barium. At this point, air may also be pushed into the stomach using a tiny tube. Thereafter, many X-ray images are captured. This outlines any abnormal spots in the lining of these organs since X-rays cannot penetrate the covering of barium. This X-ray examination examines the esophagus, stomach, and first segment of the small intestine's inner lining. Since it can miss certain suspicious regions and prevents the clinician from taking biopsy samples, this test is less frequently performed than upper endoscopy to check for stomach cancer or other issues [42].

Treatment options for *H. pylori *and gastric carcinoma

*H. pylori* infection is treated using different types of antibiotics regimens. First-line therapy includes proton pump inhibitors, clarithromycin, and amoxicillin or metronidazole. Second-line therapy is bismuth-containing quadruple therapy, which includes proton pump inhibitors, bismuth salt, tetracycline, and metronidazole [43].

Various treatment options available for gastric carcinoma are surgery, endoscopic mucosal resection, chemotherapy, radiation therapy, chemoradiation, and immunotherapy [43].

Surgery

A subtotal gastrectomy involves the removal of part of the stomach having cancer and any other organs and tissues that lie in close proximity of the tumor and lymph nodes. The whole stomach, adjacent lymph nodes, portions of the esophagus, and large and small intestines are resected during a total gastrectomy procedure. The ability to swallow food is not affected because the esophagus is attached to the small intestine. Endoluminal stent implantation, Endoluminal laser therapy, and gastrojejunostomy are possible treatments if cancer is obstructing the gastrointestinal tract [43].

Endoscopic Mucosal Resection

An endoscope is a tubular device in which a light source is attached with a viewing lens. It could have instruments for taking biopsy samples in the digestive tract. Patients with early-stage cancer will benefit from the resection of the tumor using an endoscope during an endoscopic mucosal resection treatment [43].

Chemotherapy

In chemotherapy, various medicines are used in different combinations against malignant cells to stop their growth and to kill them. These medicines enter in the bloodstream after administration through the intravenous, intramuscular, or oral route and act against malignant cells is known as the systemic type of chemotherapy. Another type is the regional type, in which medicines are directly administered into the target organs. The route of administration is decided on the basis of the stage of cancer [43].

Radiation Therapy

High-energy radioactive substances and X-rays are used in radiotherapy to destroy cancer cells, stop their growth, and to reduce the size of tumors. These high-energy radiation sources damage the DNA of cancer cells and reduce their ability to grow. Radiotherapy acts slowly on cancer cells by diminishing their ability to grow and reproduce [43].

Prevention

Behavioral changes are the effective primary prevention methods. Prevention is possible through dietary intervention. This involves the consumption of more fruits, alliums, plenty of whole grain food,s like cereals and breads, and non-starchy vegetables. Less consumption of salt, salt-preserved foods like pickles, salted meats and fish, and N-nitroso substances are beneficial against the disease [38]. Maintaining a healthy weight, quitting smoking, and total abstinence from alcohol can lower the risk of developing the illness [39]. The inclusion of foods containing antioxidants in the diet is crucial in preventing the emergence of cancer. The active ingredient in red fruits and vegetables is lycopene. Lycopene-rich food consumption significantly reduces the risk of GC, and its ingestion decreases the risk of various malignancies. Lycopene reduces the formation of reactive oxygen species, which prevents the proliferation of cancer cells. *H. pylori* disease can linger for years and gradually advance from preneoplastic lesions to GC. Eliminating the infection may be the most effective way to prevent this disease. Eradicating* H. pylori* leads to a reduction in the recurrence rate of early gastric cancer and suppresses the recurrence of peptic ulcers [44].

## Conclusions

A key contributor to the growth of gastric carcinoma is *H. pylori* disease. Relationships between virulence factors of *H. pylori* and related outcomes, particularly GC, have brought attention to the importance of taking considerations into account when predicting disease outcomes. In Western nations, having CagA increases the likelihood of developing a serious illness. Over decades, *H. pylori *disease and carcinogenesis have been caused by factors like bacterial virulence, host genetics mechanism, and environmental variables. Oral microbes play an important role in the pathogenesis of gastric cancer by various mechanisms like suppression of the immune system of the host, anti-apoptotic activity, and chronic inflammation. Risk factors like family history, salt intake, and smoking play a crucial role in the progression of the disease. Diagnostic tests like urea breath test, *H. pylori* antigen testing, and stool antigen testing are helpful in diagnosing the disease. For the diagnosis of gastric cancer, imaging modalities like endoscopic ultrasound, CT scan, MRI, and PET scans are useful. Treatment options for gastric cancer are radiation therapy, chemotherapy, surgery, and endoscopic mucosal resection. Antibiotics and antimicrobials are used for the treatment of *H. pylori* infection. Systematically eliminating carcinogenic *H. pylori* from a high-risk population will significantly lower the occurrence of stomach carcinoma globally. Lifestyle modifications and dietary regulations are essential factors in preventing the disease. Eliminating *H. pylori* in people may reduce their risk of acquiring stomach cancer. When eradicating *H. pylori* in the common population, consideration should be given to whom to kill, when to eliminate, and what regimen to apply.
